# Conditional dependence of the “male–SCC, female–ADC” teaching heuristic in lung cancer: shifts in exposure pathways and the need for pathology education reform

**DOI:** 10.3389/fpubh.2026.1847300

**Published:** 2026-06-26

**Authors:** Li-Nan Chen, Mu-Wei Dai

**Affiliations:** 1 Department of Pathology, Huantai County People's Hospital, Zibo, Shandong, China; 2 Department of Orthopedics, The Fourth Hospital of Hebei Medical University, Shijiazhuang, Hebei, China

**Keywords:** adenocarcinoma, histological subtype, lung cancer, medical education, PM2.5, public health, squamous cell carcinoma

## Abstract

The classic pathological teaching aphorism that “male lung cancer is predominantly squamous cell carcinoma (squamous cell carcinoma, SCC), whereas female lung cancer is predominantly adenocarcinoma (adenocarcinoma, ADC)” remains widely used in medical education, yet it is now markedly discordant with contemporary epidemiological evidence. We argue that this discrepancy does not indicate that the aphorism was historically incorrect; rather, the three exposure premises on which it was originally founded have been fundamentally altered in the modern era. The substantial global decline in male smoking prevalence has weakened the population basis of SCC, cigarette filter ventilation design, as a hypothetical contributing factor, has altered the intrapulmonary deposition pattern of inhaled carcinogens, while fine particulate matter (PM2.5) can activate pre-existing driver mutations in lung epithelial cells through inflammatory signaling, thereby establishing a tobacco-independent carcinogenic pathway favoring ADC. The convergence of these exposure shifts has enabled ADC to replace SCC as the most common lung cancer subtype in both men and women across most countries and regions. However, the teaching aphorism has never been explicitly annotated with its historical conditional boundaries. As a result, a screening cognition framework still centered on the “smoking–SCC” paradigm may generate substantial blind spots in clinical practice, potentially contributing to missed detection and diagnostic misclassification. We therefore advocate replacing this static mnemonic with a conditional and dynamic teaching framework structured around temporal, exposure, and population dimensions, and call for prioritizing risk-stratified lung cancer screening strategies for never-smokers together with individual-level causal validation of the PM2.5-driven carcinogenic pathway.

## 1 Introduction

In pathology and internal medicine teaching across most medical schools worldwide, the aphorism that “male lung cancer is predominantly squamous cell carcinoma (squamous cell carcinoma, SCC), whereas female lung cancer is predominantly adenocarcinoma (adenocarcinoma, ADC)” is still commonly transmitted as a conventional pathological teaching point. However, this educational convention is now markedly inconsistent with modern epidemiological evidence: the incidences of SCC and small cell lung cancer have continued to decline (1), whereas the incidence of ADC has progressively increased, making it the most common histological subtype of lung cancer in both men and women (2). This discrepancy reflects a broader structural deficiency in oncology education, namely fragmented curricular organization and delayed knowledge updating (3).

This does not mean that the traditional textbook view was historically incorrect; rather, its validity was contingent upon a specific exposure landscape. Before the 1990s, under conditions characterized by high smoking prevalence and conventional cigarette design, SCC was indeed the dominant histological subtype. Thereafter, the combined effects of changing smoking behaviors, alterations in cigarette composition, and increasing environmental air pollution progressively shifted the epidemiological pattern, allowing ADC to become the leading subtype in multiple countries, including the United States and Canada (4, 5).

However, this aphorism has never been explicitly annotated with its historical conditional boundaries, thereby fostering a cognitive anchoring bias around the “sex–subtype” correspondence (6). Clinicians may consequently systematically underestimate the risk in patients who do not conform to this paradigm (7), potentially leading to missed screening opportunities and delayed diagnosis (8). Cognitive bias is already recognized as one of the principal contributors to diagnostic error: in the United States alone, approximately 795,000 individuals each year experience permanent disability or death due to misdiagnosis of dangerous diseases, and lung cancer ranks among the five conditions associated with the greatest severe harm (9). This underscores the public health cost that cognition-driven diagnostic bias can impose, and suggests that a mismatch between pathological teaching heuristics and contemporary epidemiological reality may generate comparable downstream consequences that warrant urgent attention.

The risk generated by this outdated aphorism does not directly interfere with authoritative contemporary diagnostic procedures such as histopathology or molecular testing; rather, it exerts its influence on upstream stages of the diagnostic pathway, including pre-visit risk appraisal, primary care triage, referral thresholds, and screening recommendations. For physicians whose pathological cognition remains anchored to the “male–smoker–SCC” paradigm, the lung cancer risk of a never-smoking woman with long-term PM2.5 exposure may be systematically underestimated, potentially delaying imaging referral and consequently postponing the downstream diagnostic process (8), with adverse clinical implications.

**Figure 1:**
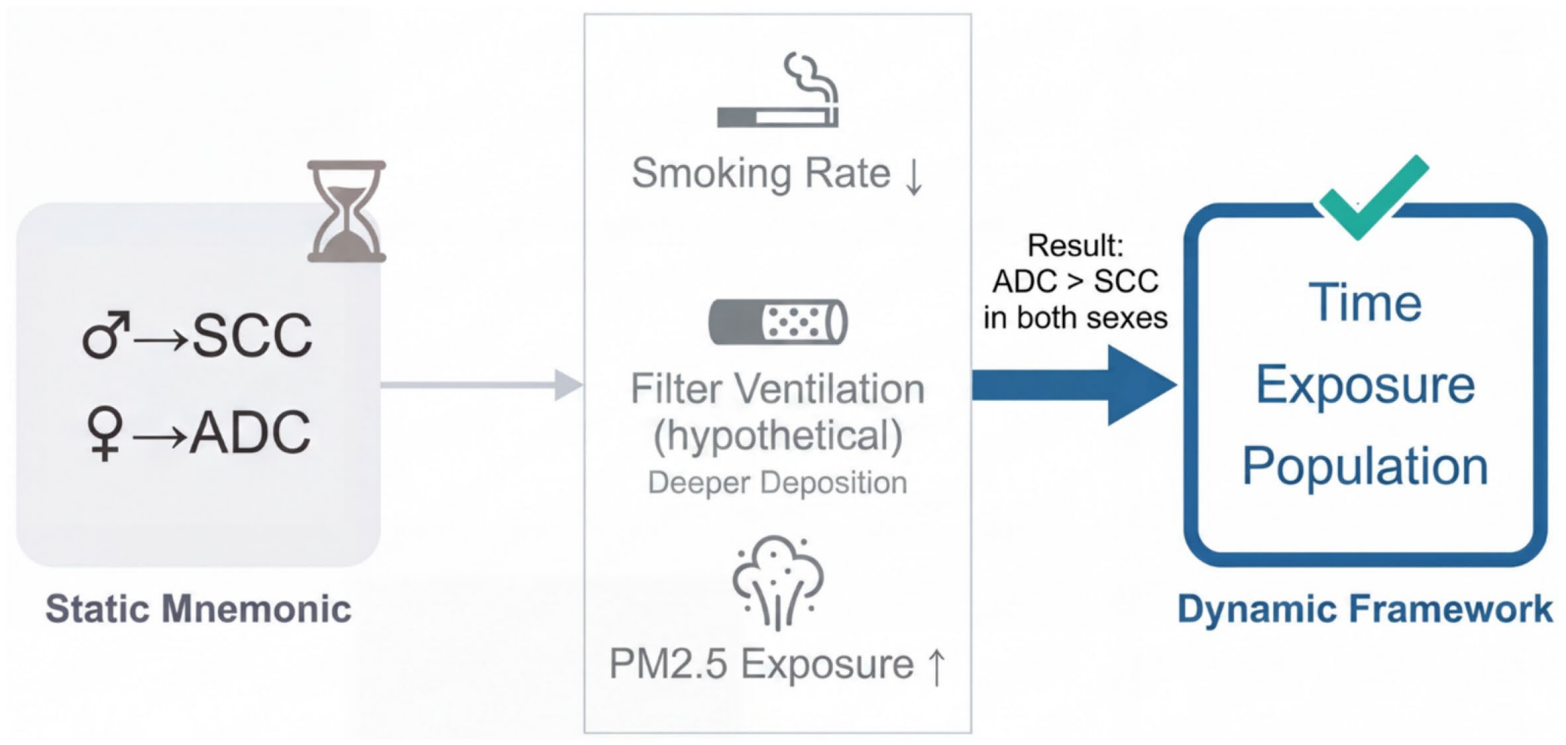
Beyond “Men with SCC, Women with ADC”: An exposure-driven conditional framework for lung cancer histological subtypes. This figure illustrates, in a unidirectional left-to-right logical sequence, the paradigm shift in pathological teaching of lung cancer.

We therefore argue that a pathological teaching aphorism that no longer accords with epidemiological reality is not merely a matter of academic inaccuracy, but a direct and underrecognized source of public health risk (Figure 1).

## 2 Divergence between textbook heuristics and contemporary reality

In the current 10th edition (2024) of the nationally planned five-year undergraduate medical textbook *Pathology* in China, SCC is still described in the lung cancer chapter as a centrally located tumor “more common in men and closely associated with smoking,” whereas ADC is described as a peripheral tumor “more common in women” (10). This traditional cognition—that male lung cancer is mainly SCC whereas female lung cancer is mainly ADC—largely originated in the mid-20th century, when male smoking prevalence was far higher than that of females and smoking showed a particularly strong association with centrally located SCC, thereby giving rise to the simplified experiential conclusion of “men with SCC, women with ADC” (11). This is not an isolated example. A similar pathological framing is also retained in the etiological section of the 10th edition (2024) of the nationally planned undergraduate medical textbook *Internal Medicine* in China (12).

This aphorism emerged from a highly specific epidemiological and historical background. Temporally, it was mainly applicable from approximately 1950 to the early 1990s, corresponding to the period when cigarette consumption among men in high-income countries reached its peak, ventilated filter cigarettes had not yet been widely adopted, and male smoking prevalence had not substantially declined (13, 14). Geographically, it was chiefly applicable to regions with the most complete cancer registry documentation, including North America, Northern and Western Europe, and East Asia (15). Population-wise, it most closely matched heavy-smoking men exposed to non-ventilated, high-tar cigarettes. This pattern is clearly reflected in the United States SEER-9 registry data from 1973 to 2010: SCC remained the most common histological subtype among male lung cancers from 1973 onward, accounting for approximately half of male lung cancer cases during the 1970s, whereas ADC represented only about 20% in 1973; thereafter, the relative proportion of SCC steadily declined to roughly 30%, while ADC progressively rose to exceed 40%, ultimately becoming the most common histological subtype in both sexes (16). Similar evidence has been documented in Europe. Charloux et al., based on registry data from the United Kingdom, France, Nordic countries, and other European regions, likewise recorded a male histological distribution dominated by SCC before the late 1980s (17). We therefore contend that what this aphorism encoded was not an erroneous empirical summary, but an epidemiological regularity tightly bound to a specific temporal window, a specific exposure configuration, and a specific population, and thus one whose scope of applicability requires strict qualification rather than unconditional inheritance as a timeless rule.

Today, the exposure conditions have fundamentally diverged from those of the past, including declining smoking prevalence, changes in cigarette design, and rising PM2.5 burden (13, 18, 19). Under these modern conditions, the once-valid pathological heuristic has acquired substantial limitations and is now sharply inconsistent with the current epidemiological reality in which ADC accounts for 52.4% of all lung cancers and has surpassed SCC to become the most common subtype even among men (16, 19). This educational lag does not directly affect final histological or molecular diagnosis, but rather influences the upstream stages of diagnostic reasoning—particularly risk appraisal and screening decision-making—where cognitive anchoring bias is most likely to arise (6, 8).

Yet this outdated view continues to be reinforced through educational inertia, which may cause medical students to develop a perception of contemporary lung cancer subtype distribution that is increasingly detached from reality. At the same time, broader deficiencies in oncology education, including curricular fragmentation and molecular testing training completion rates of ≤10%, may further aggravate this disconnect (20, 21). Although calls for curricular reform have persisted, actual progress remains notably slow (3), and the pathological teaching content related to lung cancer is in particular need of urgent updating.

## 3 Profound shifts in the underlying exposure landscape

The validity of the “men with SCC, women with ADC” pathological conclusion depended on three principal exposure premises: a high smoking prevalence among men, concentrated deposition of carcinogens in the central airways from traditional non-ventilated cigarettes, and relatively low levels of environmental exposure such as PM2.5. However, these conditions no longer exist across most countries and regions today (13, 18, 19).

With respect to smoking prevalence, the global advancement of tobacco control has produced a sustained downward trend. In most high-income countries, male smoking prevalence has declined continuously from its mid-20th century peak (14) to generally below 25% by 2019 (15). This directly weakened the population basis for squamous cell carcinoma and coincided with a global epidemiological pattern in which adenocarcinoma incidence has surpassed SCC in men across 150 countries and in women across all 185 surveyed countries (22). Among the approximately 2.48 million newly diagnosed lung cancer cases worldwide in 2022, ADC accounted for 52.4% overall; among men, ADC represented 45.6% compared with 29.4% for SCC, whereas among women ADC accounted for 59.7% and SCC only 17.1% (19). Under such circumstances, the pathological teaching notion that “male lung cancer is mainly SCC” is no longer tenable in most countries. Notably, in specific regions such as northern India, where bidi tobacco use remains highly prevalent and diagnostic capacity differs, SCC may still predominate among male smokers (23), which further indirectly confirms that high male smoking prevalence constitutes the demographic foundation of this traditional pathological view.

This histological transition has also been validated to a considerable extent by international cancer registry data. An international comparative analysis based on data from 12 population-based registries across 11 countries covering the period from 1973 to 2002 demonstrated that the incidence of male ADC rose continuously and surpassed the historically dominant SCC in most study populations, with only North America, Australia, and Iceland showing signs of ADC plateauing by the mid-1980s (4). Likewise, in European populations, ADC has overtaken SCC in both men and women across the majority of countries (17, 22), with the transition being particularly pronounced in Northern and Western Europe.

Second, changes in cigarette design represent a second major, although still hypothetical, contributing factor to this histological redistribution. Available evidence suggests that filter ventilation holes may alter the physical characteristics of mainstream smoke, allowing smaller tobacco-specific carcinogenic particles, including nitrosamines such as NNK, to bypass mucociliary clearance in the central airway, penetrate more deeply, and deposit in the peripheral alveolar–bronchiolar junction where ADC preferentially arises (13). However, the magnitude of this cigarette-design effect and its quantitative contribution to the population-level increase in ADC remain controversial (24). This is largely because current evidence is dominated by observational rather than experimental studies, while substantial cross-national differences also exist in cigarette regulatory frameworks: the U.S. Food and Drug Administration operates under the Family Smoking Prevention and Tobacco Control Act, the European Union implements the Tobacco Products Directive, and many Asian countries maintain their own tar/nicotine upper-limit standards. These differences suggest that the filter-ventilation hypothesis deserves serious consideration, but its universality should be interpreted cautiously (13, 14).

In addition, although the 2011 revision of the IASLC/ATS/ERS classification and the widespread adoption of immunohistochemical markers such as TTF-1 and p40 did lead to reclassification of some tumors previously labeled as “large cell carcinoma” or “NSCLC-NOS” into adenocarcinoma, the trend of ADC surpassing SCC had already emerged in multiple countries before this diagnostic revision (16, 17), remained equally evident among never-smokers (25), and in fact the rise in ADC proportion among never-smokers preceded the broad adoption of low-tar cigarettes (24). This indicates that diagnostic reclassification cannot be regarded as the primary driver, whereas environmental exposures such as PM2.5 are more likely to represent an important force independent of smoking behavior and cigarette design (18, 26). Globally, never-smokers account for approximately 15–25% of all lung cancer cases, of which 60–80% are adenocarcinoma (26). Experimental evidence has shown that PM2.5 can promote the occurrence and progression of EGFR-mutant lung cancer through the AhR–TMPRSS2–IL18 signaling axis (27), while a large multinational 2023 Nature PM2.5–EGFR Lung Cancer Study demonstrated a significant association between PM2.5 exposure levels and the incidence of EGFR-driven lung cancer (18).

Mechanistically, PM2.5 induces infiltration of pulmonary interstitial macrophages and stimulates IL-1β release, while promoting the progenitor-like transition of alveolar type II epithelial cells (ATII) carrying pre-existing EGFR driver mutations, thereby accelerating adenocarcinoma initiation and progression (18). This indicates that PM2.5 does not primarily generate new mutations; rather, it activates pre-existing driver alterations already present in histologically normal lung tissue through inflammatory signaling, with approximately 18% of normal lung tissue harboring EGFR driver mutations and 53% harboring KRAS mutations (18). PM2.5 is therefore more appropriately conceptualized as a promoter of lung adenocarcinogenesis rather than a classical mutagenic initiator.

Beyond the molecular level, the preferential induction of peripheral lung cancer by PM2.5 is also related to systematic differences in anatomy, particulate deposition location, and molecular susceptibility. SCC originates mainly from basal cells of the proximal conducting airway, whereas ADC arises predominantly from alveolar type II epithelial cells (AT2) in the peripheral airway and Clara cells at the bronchioalveolar junction (26, 28). Inhaled particles deposit according to aerodynamic diameter: coarse particles mainly impact the central airway, whereas PM2.5 and ultrafine particles penetrate into terminal bronchioles and alveolar regions (13). Meanwhile, peripheral AT2 cells intrinsically harbor a substantial burden of pre-existing EGFR and KRAS driver mutations (18). The interaction of these three dimensions means that dust exposure reaches the pulmonary peripheral compartment that is anatomically deepest, kinetically most accessible, and molecularly most susceptible, thereby preferentially promoting the development of peripheral lung cancer and adenocarcinoma.

Taken together, whether viewed from the perspective of declining male smoking prevalence, changing cigarette design, or increasing PM2.5 exposure, all lines of evidence indicate that the traditional teaching aphorism of “men with SCC, women with ADC” has become substantially outdated. Importantly, a 2025 study including 997 patients with lung cancer reported that approximately 65% of patients did not meet current U.S. Preventive Services Task Force screening criteria; among this non-eligible group, women, Asians, and never-smokers were significantly overrepresented, and adenocarcinoma accounted for 72% of cases compared with 55% in the eligible group (29). Such lagging pathological cognition may therefore contribute to under-screening and missed diagnosis of lung cancer, ultimately producing adverse public health outcomes.

## 4 Discussion

Given that the traditional pathological teaching view of lung cancer has never been annotated with its historical conditional boundaries, the most direct correction is not to discard it, but to conditionally refine it: “under the exposure conditions of the mid-20th century, characterized by high smoking prevalence and non-ventilated cigarettes, the proportion of SCC in men exceeded that of ADC; however, in most countries worldwide today, ADC has become the most common subtype in both men and women (19, 22).” At the same time, in response to the urgent calls for reform in current oncology education (3, 20, 21), we propose that pathology curricula incorporate three explicit instructional dimensions—temporal dimension (mid-20th century versus the contemporary era), exposure dimension (smoking prevalence/cigarette design/environmental PM2.5), and population dimension (heavy smokers/light smokers/never-smokers)—to address the growing mismatch between pathological cognition and current lung cancer epidemiology.

This approach may draw upon empirical educational pathways showing that multi-context exposure facilitates the transfer of foundational scientific knowledge (30), thereby training medical students to formulate pathological regularities as conditional propositions linked to time, exposure, and population, rather than as unconditional “laws” in the traditional pedagogical style. In parallel, the design should also follow a spiral curricular architecture (31), enabling students to revisit similar condition-dependent concepts repeatedly across different academic years, reinforce the internalization of the “pattern–condition” coupling, and establish a routine 3–5-year revision cycle (32) aligned with the evolving update rhythm of the World Health Organization lung tumor classification system (33), so as to maintain synchrony with current scientific understanding and avoid severe divergence between teaching and reality. In addition, a horizontal–vertical integration framework (34) may be adopted to define explicit curricular linkage nodes with adjacent domains such as molecular diagnosis, screening recommendations, and public health, thereby ensuring that the updated pathological knowledge can be structurally accommodated within the curriculum (35) and more effectively translated into improved physician decision-making capacity.

The 2025 Stanford Lung Cancer Summit highlighted that healthcare providers still possess insufficient awareness of lung cancer in never-smokers, while standardized screening guidelines for this population remain lacking (36). Meanwhile, the TALENT study from Taiwan provided important evidence supporting low-dose CT screening in high-risk never-smoker populations, with a baseline detection rate of 2.6%, of which 96.5% were stage 0/I disease (37). This indicates that beyond educational content itself, public cognition and screening coverage for never-smoker lung cancer also require urgent updating. However, such optimization does not imply unconditional expansion of screening to all non-smokers; rather, it calls for risk-stratified selective screening in high-risk populations while balancing multiple constraints. In the National Lung Screening Trial, approximately 96.4% of positive screening results among high-risk smokers were still benign (38). Because the baseline incidence in never-smokers is lower, the expected positive predictive value would be even poorer, magnifying the burden of false positives, overdiagnosis, and invasive downstream procedures, while also encountering limitations in quality-adjusted life years and incremental cost-effectiveness ratios. At the same time, the eligibility threshold should refer to recommendations from the International Association for the Study of Lung Cancer Early Detection and Screening Committee (39), integrating PM2.5 exposure, family history, secondhand smoke, and occupational exposure rather than relying solely on smoking history, although such a multidimensional model would inevitably increase economic cost and data complexity. Moreover, special stratified consideration is warranted because the TALENT cohort was enriched for family history and existed in a relatively high PM2.5 background (37).

In addition, the critical knowledge gaps necessary to support both educational and screening reform must be filled in a timely manner. Given that the temporal window and dose–response characteristics of the PM2.5–IL-1β–EGFR promotional carcinogenic axis remain insufficiently characterized (18), future studies should prioritize large multicenter prospective cohorts to validate, at the individual level, the causal relationship between environmental exposure and adenocarcinoma subtype distribution, and to develop integrated exposure-scoring models for risk stratification in high-risk never-smoker populations (18, 36), thereby providing an evidence base for both teaching reform and screening redesign.

For end-to-end quantification of this teaching-to-screening pathway, the Kirkpatrick Four-Level Model may serve as a useful reference (40) to construct a pathology-specific four-layer assessment framework consisting of “cognitive recalibration–decision correction–clinical behavior–screening outcome.” At the cognitive level, empirical evidence regarding the influence of anchoring bias on clinical decision-making can provide baseline support (8). At the decision and behavioral levels, analogous studies may be modeled after findings showing that physicians aware of U.S. Preventive Services Task Force recommendations were significantly more likely to order low-dose CT (71% vs. 38%, *p* < 0.001) and initiate screening discussions (86% vs. 62%, *p* < 0.001) (41). At the outcome level, data capturing the mismatch between formal screening criteria and the real-world high-risk population (29) may be collected to reflect the downstream coverage gap generated by upstream cognition. Finally, prospective pre- and post-curricular update comparative studies can be used to complete the response-level evidence across all layers, thereby enabling end-to-end experimental quantification of the contribution of conditionalized teaching and filling the current gap in medical education research in this area.

## 5 Conclusion

The traditional pathological view of “men with SCC, women with ADC” in lung cancer is not a historically false description, but rather an expired conditional proposition. Adenocarcinoma has now replaced squamous cell carcinoma as the most common lung cancer subtype worldwide, and continuing to teach the old aphorism as an unconditional law itself constitutes a pedagogical bias inconsistent with contemporary evidence. We contend that updating educational content is not only a requirement of academic accuracy, but also a prerequisite for effective public health action, because accurate disease cognition is the first step toward sound risk assessment, screening, and prevention strategies.

Left panel (static mnemonic framework): Traditional clinical teaching uses sex as the primary predictor of histological subtype, thereby generating the static mnemonic that “men → squamous cell carcinoma (SCC), women → adenocarcinoma (ADC).” The hourglass symbol indicates that the historical epidemiological conditions supporting this aphorism—approximately 1950–1990, characterized by high smoking prevalence, non-ventilated cigarettes, and low PM2.5 exposure—have progressively expired over time.

Middle panel (exposure-pathway catalytic variables): Three major structural changes have reshaped the population exposure profile. (1) Overall smoking prevalence has continuously declined, as confirmed by multiple national cancer registries and GBD 2019 data. (2) The widespread adoption of low-tar ventilated-filter cigarettes has been hypothesized to promote deeper deposition of smoke particles and alter the principal airway target zone of carcinogenic injury; the label “hypothetical” indicates that this mechanism is currently supported mainly by observational evidence, that its quantitative contribution to the population-level rise in ADC remains under debate, and that cigarette design regulatory frameworks differ across jurisdictions (e.g., U.S. Food and Drug Administration in the United States and the Tobacco Products Directive in Europe). (3) Indoor and outdoor PM2.5 environmental exposure has risen substantially and functions as a promoter, rather than a classical mutagenic initiator, in lung adenocarcinogenesis by activating pre-existing driver mutations in lung epithelial cells through inflammatory signaling, thereby becoming an independent driving force for never-smoking-related ADC.

Right panel (dynamic conditional framework): The synergistic evolution of these three exposure pathways has resulted in adenocarcinoma (ADC) surpassing squamous cell carcinoma (SCC) in incidence in both men and women, rendering sex no longer a reliable predictor of histological subtype distribution. Contemporary teaching should therefore shift toward a dynamic conditional paradigm centered on Time, Exposure, and Population, so as to more accurately reflect the true evolutionary trajectory of lung cancer epidemiology.

## Data Availability

The original contributions presented in the study are included in the article/supplementary material, further inquiries can be directed to the corresponding author.
